# The role of school physical education on adolescents' fitness levels during the pandemic period from COVID-19: An observational study of the Italian scientific high school—section sport and physical activity

**DOI:** 10.3389/fpubh.2022.1010236

**Published:** 2022-09-15

**Authors:** Antonino Patti, Valerio Giustino, Flavia Figlioli, Matteo Miceli, Martina Barca, Patrik Drid, Antonio Palma, Antonino Bianco

**Affiliations:** ^1^Sport and Exercise Sciences Research Unit, Department of Psychology, Educational Science and Human Movement, University of Palermo, Palermo, Italy; ^2^Faculty of Sport and Physical Education, University of Novi Sad, Novi Sad, Serbia

**Keywords:** school, physical education, physical activity, Handgrip strength, vertical jump height, vertical jump performance, jumping performance, body posture

## Abstract

**Objectives:**

In Italy, in 2013, a new school curriculum with a sport character was established in high schools, called Sports High School (SHS). The aims of this study were: (1) to assess the fitness levels of SHS students who, respecting all the safety rules to limit the spread of COVID-19, practiced physical education (PE) at school with continuity for almost all of 2021, and to compare them with Traditional High School (THS) students; (2) to evaluate as the SHS may have influenced the fitness levels in adolescents.

**Methods:**

This is a case-control study in which thirty participants were enrolled (SHS: *n* = 15; THS: *n* = 15). To assess the fitness levels, the following tests were administered: the Static Baropodometric and Stabilometric Analyses, the Counter Movement Jump (CMJ), and the Handgrip test. All these tests were administered when the non-pharmaceutical interventions (NPIs) for COVID-19 allowed the resumption of PE lessons (T0) and 2 months after their resumption (T1).

**Results:**

Unpaired *t*-test between SHS (T0) vs. THS (T0) showed significant differences between: Handgrip test Dx and Handgrip test Sx (both *p* < 0.001), Surface Sx foot and Surface Dx foot (both *p* < 0.05), and CMJ (*p* < 0.001). These results were also confirmed in T1. The performance analysis between T0 and T1 of both SHS and THS showed improvements in SHS: Handgrip test Dx (*p* < 0.05; *d* = 0.57), Handgrip test Sx (*p* < 0.01; *d* = 0.87), and CMJ (*p* < 0.05; *d* = 0.59). Pearson's analysis of the results of the tests showed significant strong correlations between: Handgrip test Dx and Handgrip test Sx (*R* = 0.959; *p* < 0.001), Handgrip test Dx and CMJ (*R* = 0.881; *p* < 0.001), Handgrip test Sx and CMJ (*R* = 0.893; *p* < 0.001). The same analysis showed significant but moderate correlations between: Surface foot Sx and CMJ (*R* = 0.489; *p* < 0.01), Surface foot Sx and y-mean (*R* = 0.485; *p* < 0.01), Surface foot Dx and CMJ (*R* = 0.444; *p* < 0.05).

**Conclusions:**

This study is in agreement with the literature showing that the quarantine period and the NPIs for COVID-19 caused a decrease in fitness levels in adolescents. Our results showed that students of SHS recorded higher strength performance both in the Handgrip test and in the CMJ.

## Introduction

In Italy, the objectives of school education are defined by the National Guidelines. Physical activity (PA) is related with several health benefits and is widely recognized as an essential determinant of adolescents physical and psychosocial health and wellbeing (1, 2). According to these National Guidelines, the general aim of the school is the harmonious and comprehensive development of the individual (3). In Italy, in 2013, a new school curriculum with a sport character was established in high schools, called Sports High School (SHS) (4). This curriculum is aimed at the study of sports sciences and PA. In addition, unlike the traditional curriculum, that is Traditional High School (THS), it provides for the practice of sport and PA and from 2 to 6 h weekly in the first 2 years, and 5 h in the last 3 years, significantly increasing the annual hours that students dedicate to physical education (PE) at school.

In 2019, to counteract the coronavirus disease-2019 (COVID-19), the Italian government adopted containment measures to control the virus' spread known as non-pharmaceutical interventions (NPIs). Among the NPIs, schools and SHS have been closed and PE have been suspended (5). Even when school activity returned to normal, PE continued to have severe limitations throughout 2021. However, in the same year, the Italian Ministry of Public Education issued a clarification regarding the specific skills that can only be achieved through regular and constant practice (6). In particular, at the end of the 5-year period, students in SHS must acquire the specific knowledge and skills of the various individual and team sports envisaged by their characterizing school curriculum. To this end, these students have had priority in accessing school sports facilities while respecting social distancing and contingency of sports spaces (7).

The literature suggests that schools play an essential role in combating physical inactivity and in ensuring good health (1). In 2016, the World Health Organization (WHO) identified schools as the optimal context to implement and guarantee adequate levels of PA (8). Adequate levels of PA are considered to be at least 60 min per day, most of this PA should be aerobic (9).

These unique circumstances have allowed us to study some aspects of the influence of the PE programs of the Italian school. The purpose of this study was double: (1) the first aim was to assess the fitness levels of SHS students who, respecting all the safety rules to limit the spread of COVID-19, practiced PE at school with continuity for almost all of 2021, and to compare them with THS students who have experienced greater limitations in practicing PE as a result of the restrictions; (2) analyzing a 2-month of follow-up, the second aim was to evaluate as a specific school curriculum aimed at the study of sports sciences and PA, that increases the annual hours that students dedicate to PE at high school, may have influenced the fitness levels in young adolescents compared to students of traditional school curriculum. To the best of our knowledge, this is the first study that evaluated the effects of this specific school curriculum, established in Italy in 2013 with the name of “Scientific high school—section sport and physical activity” (4).

## Materials and methods

### Study design and procedure

This is a case-control study in which participants were students of the same high school located in Sicily Region (Italy). Participants of the SHS group were part of a high school class with a sport curriculum, while the THS group was composed of students attending classes with a traditional PE curriculum. Written informed consent was obtained before participating in the study from parents' participants. The STROBE guidelines were used to ensure a high-quality presentation of the conducted observational study (10). The study was carried out in compliance with the principles of the Declaration of Helsinki and approved by the Bioethics Committee of the University of Palermo (Num. 77/2022 - Prot. 35307).

### Participants

**Figure 1:**
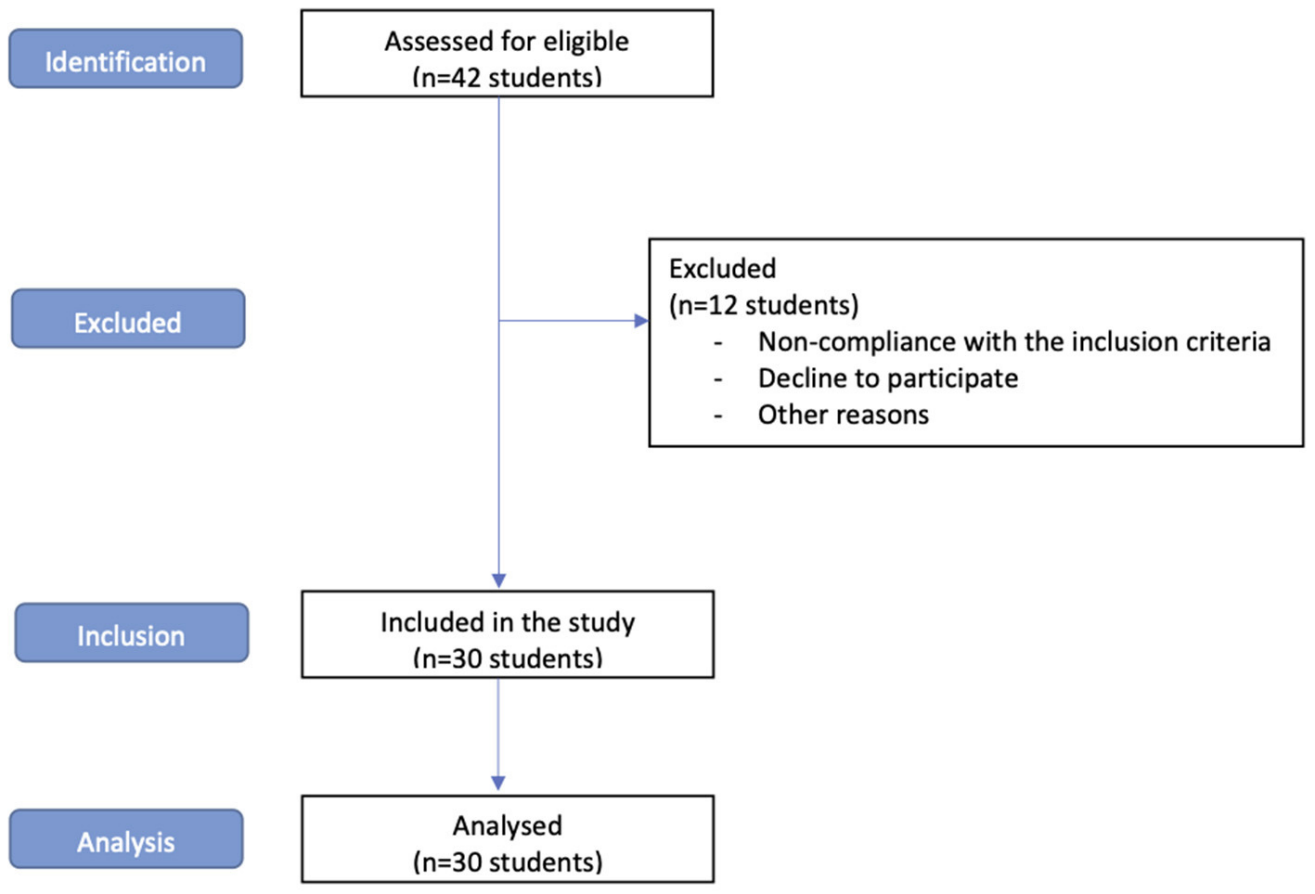
STROBE flow chart.

Forty-two subjects were considered for the study but twelve of them did not meet the inclusion criteria or declined their participation. Hence, thirty subjects were enrolled in this study (Figure 1). Fifteen attended classes with a sport curriculum and fifteen attended classes with a traditional PE curriculum. To be eligible for the study, students met the following inclusion criteria: (a) they did not have to play professional sports or structured PA in sports clubs during after-school hours; (b) they were not have any deficits or illnesses affecting sports and fitness performance; (c) during the pandemic period they had not carried out any PA with sports professionals other than those activities authorized by the school.

### Sports high school vs. traditional high school

The SHS was established in Italy in 2013 with the name of “Scientific high school—section sport and physical activity” (4). The SHS is a 5-year upper secondary school that combines the theme of the traditional Italian scientific high school with a substantial increase in the hours of lessons dedicated to PE and the study of sport and PA in general. This topic is also dealt in other disciplines, such as Sports Law and Economics. The SHS aims to combine an in-depth and harmonious culture, both in the humanistic and scientific fields, through the promotion of the educational value of sport. This specific high school is characterized by the strengthening of physical and sports sciences. In particular, at the end of the course, students who attend a scientific high school with a sport curriculum must: (a) know how to apply the methods of sport practice in various fields, (b) know how to elaborate the critical analysis of sport, the methodological on sport and the experimental procedures on it, (c) know how to search for strategies aimed at promoting the discovery of the multidisciplinary and social role of sport, (d) knowing how to deepen the knowledge and practice of the various sports (4). In detail, in the first 2 years, the students of this curriculum dedicate 3 h per week to PE and further 3 h dedicated to specific sports activities. In the following 3 years, the hours dedicated to PE became two per week while those dedicated to specific sports activities remained three. On the other hand, the traditional curriculum of Italian high schools provides only 2 h per week of PE.

### Measurements

To assess the fitness levels, the following tests were administered: the Static Baropodometric and Stabilometric Analyses, the Counter Movement Jump (CMJ), and the Handgrip test. All these tests were administered when the non-pharmaceutical interventions (NPIs) for COVID-19 allowed the resumption of PE lessons for all students enrolled in the study (T0) and 2 months after the resumption of PE lessons in which they restarted normally and continuously for both groups (T1).

Static Baropodometric and Stabilometric Analyses were carried out using the FreeMed system (FreeStep v.1.0.3 software, Sensor Medica, Guidonia Montecelio, Roma, Italy). The platform's sensors are 24 K gold and this allows a high reliability. Using the Romberg test position, all participants were administered a baropodometric and a stabilometric analysis (11). The parameters considered were: Surface Sx foot (left), Surface Dx foot (right) for the baropodometric analysis, and Sway Path Length (mm), Ellipse Surface Area (mm^2^), x-mean (mm), y-mean (mm), and Average Speed of Movement (mm/s) for the stabilometric analysis (12).

Counter Movement Jump (CMJ) test was administered with the Microgate system. It is an optical detection system composed of a transmitting and a receiving bar. The system allows the measurement of flight and contact times during the execution of a series of jumps, with a precision of 1/1,000 of a second. Starting from these fundamental basic data, the dedicated software allows to obtain a series of parameters related to performance with maximum precision and in real-time (13).

Handgrip test aims to measure the maximum isometric strength exerted by the upper limb muscles. The literature suggests that the Handgrip test has a predictive value and correlation with the fitness levels (14–17).

### Statistical analysis

All numerical data were entered into an Excel sheet before being analyzed. Descriptive statistics were reported as mean ± standard deviation. Shapiro–Wilk's normality test was used to analyze data distribution. *Post hoc* sample size power analysis was computed to estimate the level of power achieved for the sample size using G^*^Power software 3.1.9.2 (Heinrich Heine University, Düsseldorf, Germany). The comparison of the two groups at T0 and at T1 was performed with unpaired Student's *t*-test, and Mann-Whitney test has been used when appropriate. The comparison of the same group over time (T0 to T1) was performed with paired Student's *t*-test, and Wilcoxon matched-pairs test has been used when appropriate. For each outcome, Cohen's *d* was calculated. Pearson's correlation coefficient was used to analyze any correlations between the variables of the tests. A *p*-value lower than 0.05 (95% confidence) was considered useful. Statistical analyses were conducted using Jamovi software (version 2.3.0.0) and GraphPad Prism 8.0.

## Results

**Figure 2:**
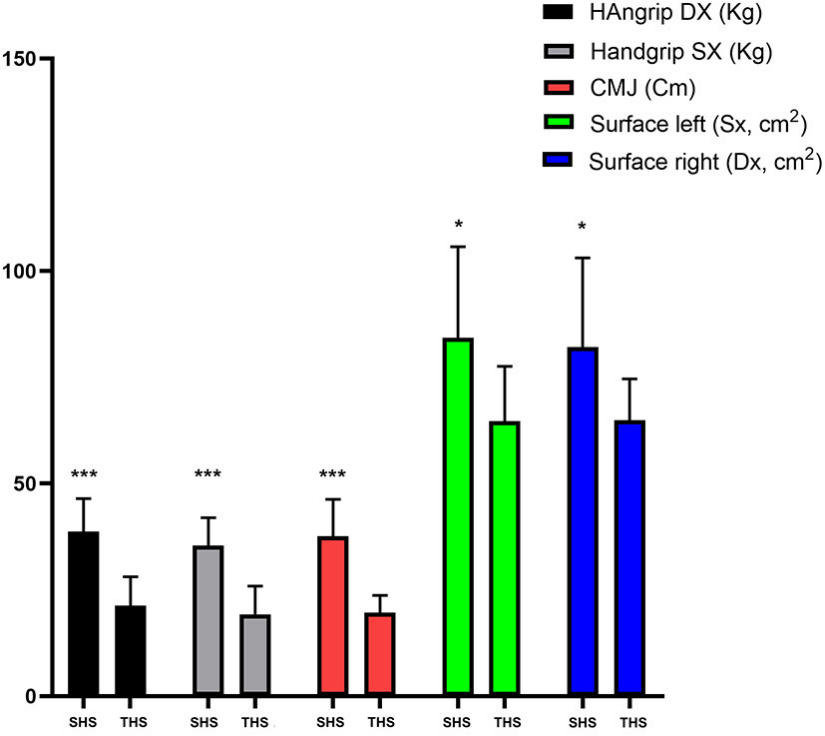
Analysis between SHS (T0) vs. THS (T0). * *p* < 0.05; ** *p* < 0.01; *** *p* < 0.001.

**Figure 3:**
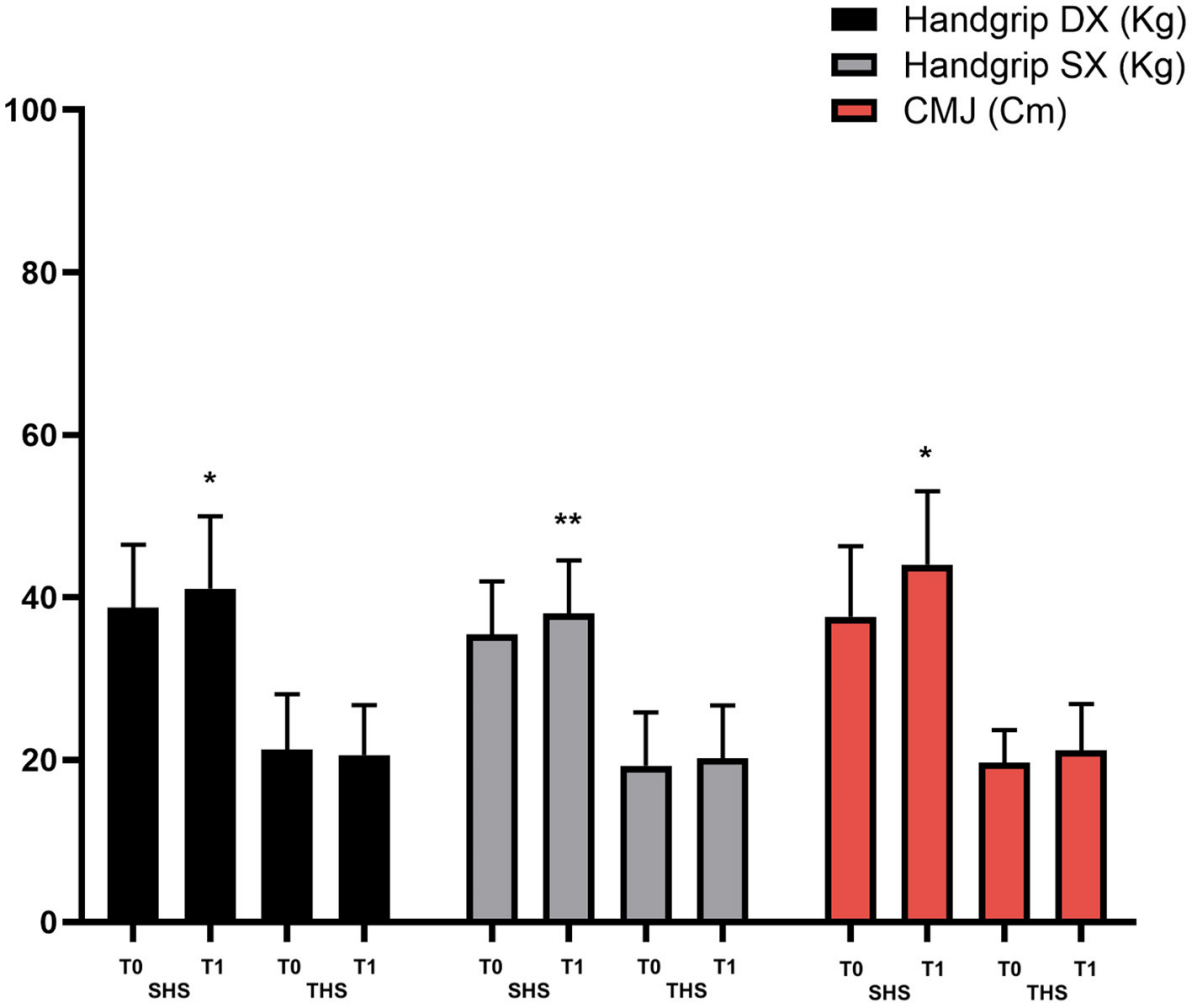
Analysis between SHS (T0) vs. SHS (T1) and THS (T0) vs. THS (T1). * *p* < 0.05; ** *p* < 0.01; *** *p* < 0.001.

*Post-hoc* sample size power analysis (*f* = 0.25, α = 0.05) showed that, with a total sample size of 30 participants (SHS: *n* = 15; THS: *n* = 15), we achieved a power of 75%. Table 1 shows anthropometric characteristics of the participants. No significant differences on anthropometric characteristics between groups were found. Table 2 and Figure 2 show unpaired *t*-test analysis between SHS (T0) vs. THS (T0). Table 3 shows the analysis between SHS (T1) vs. THS (T1). Table 4 and Figure 3 show the performance analysis between T0 and T1 of both the SHS and the THS. Pearson's analysis of the results of the tests showed significant strong correlations between: Handgrip test Dx and Handgrip test Sx (*R* = 0.959; *p* < 0.001), Handgrip test Dx and CMJ (*R* = 0.881; *p* < 0.001), Handgrip test Sx and CMJ (*R* = 0.893; *p* < 0.001). The same analysis showed significant but moderate correlations between: Surface foot Sx and CMJ (*R* = 0.489; *p* < 0.01), Surface foot Sx and y-mean (*R* = 0.485; *p* < 0.01), Surface foot Dx and CMJ (*R* = 0.444; *p* < 0.05).

**Table 1:** Unpaired *t*-test analysis of the anthropometric measures between SHS (T0) and THS (T0).

	**SHS (T0) (*n =* 15)**	**THS (T0) (*n =* 15)**	***p***
Age, y	17 ± 1.25	16.2 ± 1.42	0.14
Height, cm	167.93 ± 8.40	167.86 ± 10.41	0.98
Weight, kg	61.80 ± 9.15	61.20 ± 12.29	0.87
Shoe number	40.86 ± 2.99	40.60 ± 3.64	0.83
BMI	21.83 ± 2.07	21.59 ± 2.91	0.80
BMI Z-Score	0.11 ± 0.81	0.09 ± 1.03	0.95

SHS, Sports High School; THS, Traditional High School.

**Table 2:** Unpaired t-test analysis between SHS (T0) vs. THS (T0).

	**SHS (T0) (*n =* 15)**	**THS (T0) (*n =* 15)**	***p***	**Effect size**
				***Cohen's d***
Handgrip Dx (kg)	38.72 ± 7.74	21.26 ± 6.83	0.001	2.75
Handgrip Sx (kg)	35.44 ± 6.52	19.24 ± 6.62	0.001	2.32
Surface left (Sx, cm^2^)	84.33 ± 21.40	64.67 ± 12.93	0.05	0.75
Surface right (Dx, cm^2^)	82.13 ± 20.95	64.86 ± 9.76	0.05	0.76
CMJ (cm)	37.6 ± 8.70	19.66 ± 4	0.001	3.55
Sway path length (mm)^Φ^	402.53 ± 118.5	385.1 ± 98.85	0.6	0.12
Ellipse surface area (mm^2^)^Φ^	245.33 ± 191.89	273.94 ± 261.12	0.9	0.08
x-mean (mm)	−1 ± 13.75	−1.08 ± 12.76	0.98	0.003
y-mean (mm)	−26.95 ± 25.36	−24.51 ± 14.40	0.73	0.08
Average speed of movement (mm/s)^Φ^	8.03 ± 2.32	7.73 ± 1.99	0.86	0.11

SHS, Sports High School; THS, Traditional High School; CMJ, Counter Movement Jump; Dx, right; Sx, left; Φ, non-parametric analysis.

**Table 3:** Unpaired t-test analysis between SHS (T1) vs. THS (T1).

	**SHS (T1) (*n =* 15)**	**THS (T1) (*n =* 15)**	***p***	**Effect size**
				***Cohen's d***
Handgrip Dx (kg)	41.08 ± 8.29	20.58 ± 6.15	0.001	2.98
Handgrip Sx (kg)	38 ± 6.56	20.21 ± 6.47	0.001	2.22
Surface left (Sx, cm^2^)	89 ± 24.17	61.46 ± 9.15	0.01	0.91
Surface right (Dx, cm^2^)	83.86 ± 24.68	65.13 ± 8.99	0.05	0.67
CMJ (cm)	44 ± 9.04	21.17 ± 5.71	0.001	1.88
Sway path length (mm)^Φ^	375.60 ± 118.5	401.2 ± 103.27	0.33	0.16
Ellipse surface area (mm^2^)^Φ^	218.55 ± 186.27	231.31 ± 165.31	0.6	0.04
x-mean (mm)	−1.41 ± 12.31	0.61 ± 10.93	0.69	0.10
y-mean (mm)	−24.81 ± 21.54	−24.51 ± 14.40	0.99	0.003
Average speed of movement (mm/s)^Φ^	8.5 ± 2.84	8.49 ± 1.65	0.40	0.004

SHS, Sports High School; THS, Traditional High School; CMJ, Counter Movement Jump; Dx, right; Sx, left.

Φ, non-parametric analysis.

**Table 4:** Paired Student's *t*-test analysis between SHS (T0) vs. SHS (T1) and THS (T0) vs. THS (T1).

	**SHS (T0) (*n* = 15)**	**SHS (T1) (*n* = 15)**	***p < ***	**Effect size**	**THS (T0) (*n* = 15)**	**THS (T1) (*n* = 15)**	***p***	**Effect size**
				***Cohen's d***				***Cohen's d***
Handgrip Dx (kg)	38.72 ± 7.74	41.08 ± 8.29	0.05	0.57	21.26 ± 6.83	20.58 ± 6.15	0.48	0.18
Handgrip Sx (kg)	35.44 ± 6.52	38 ± 6.56	0.01	0.87	19.24 ± 6.62	20.21 ± 6.47	0.23	0.32
Surface left (Sx, cm^2^)	84.33 ± 21.40	89 ± 24.17	0.60	0.13	64.67 ± 12.93	61.46 ± 9.15	0.51	0.17
Surface right (Dx, cm^2^)	82.13 ± 20.95	83.86 ± 24.68	0.85	0.04	64.86 ± 9.76	65.13 ± 8.99	0.94	0.01
CMJ (cm)	37.6 ± 8.70	44 ± 9.04	0.05	0.59	19.66 ± 4	21.17 ± 5.71	0.39	0.25
Sway path length (mm) ^Φ^	402.53 ± 118.5	375.60 ± 118.5	0.48	0.16	385.1 ± 98.85	401.2 ± 103.27	0.93	0.13
Ellipse surface area (mm^2^) ^Φ^	245.33 ± 191.89	218.55 ± 186.27	0.84	0.09	273.94 ± 261.12	231.31 ± 165.31	0.56	0.16
x-mean (mm)	−1 ± 13.75	−1.41 ± 12.31	0.92	0.02	−1.08 ± 12.76	0.61 ± 10.93	0.70	0.10
y-mean (mm)	−26.95 ± 25.36	−24.81 ± 21.54	0.81	0.06	−24.51 ± 14.40	−24.51 ± 14.40	0.96	0.01
Average speed of movement (mm/s) ^Φ^	8.03 ± 2.32	8.5 ± 2.84	0.76	0.12	7.73 ± 1.99	8.49 ± 1.65	0.28	0.36

SHS, Sports High School; THS, Traditional High School; CMJ, Counter Movement Jump; Dx, right; Sx, left.

Φ, non-parametric analysis.

## Discussion

The literature suggests that physical fitness is a significant health marker already in adolescents (18).

To the best of our knowledge, there are no studies in the literature that analyzed the effects of specific school curriculum aimed at the study of sports sciences and PA on the fitness levels in adolescents. Moreover, the period in which the study was conducted allowed us to estimate the possible contribution of both this sport curriculum and the traditional PE of Italian high schools in adolescents.

In our opinion, the results of this study are particularly interesting. In fact, the main results of this study are related to the performance of SHS and THS students. Our results showed that students of SHS recorded higher strength performance both in the CMJ test and in the Handgrip test. In detail, as for the CMJ test, the THS showed significant lower values than the SHS. This result is critical for the health of adolescents. As a matter of fact, some studies described how lower limb performance is an important health marker (18–21). Indeed, it is widely known that muscle strength and cardiorespiratory endurance are considered key indicators of human health (22). In the same way, the handgrip test is used as a marker of fitness level or predictor of future health conditions. Many research groups have emphasized the work of assessing the health status of adolescents and children (11, 23). These authors developed some fitness battery tests applicable in children and adolescents as the ALPHA fitness test battery, the ASSO project, and the PREFIT (11, 14, 23, 24).

As concern the PA levels during COVID-19, of particular interest for the Italian population is the study by Giustino et al. in which authors showed significantly lower levels of PA among the physically active Sicilian population during the quarantine compared to before the quarantine at all ages (25). This trend also occurs in other countries. In fact, other research groups around the world have found a decrease in the practice of physical activity as a consequence of COVID-19 (26). Our results are in line with these studies, in fact, the students who practiced PE (SHS) have minimized the deficits in their fitness levels compared to peers who did not practice PE (THS) during the pandemic period. In fact, as abovementioned, this study allowed us to estimate the effects of school PE both in SHS and THS. In Table 4, the results showed that 2 months of traditional school PE did not improve fitness levels in adolescents. On the other hand, in the same period considered, the students who attended the SHS classes showed improved performance in handgrip test Dx, handgrip test Sx, and CMJ. Cohen's *d* analysis showed a medium/large effect size in these measures.

The limitation of this study is that we only evaluated strength and balance performance and we did not considered the assessment of cardiorespiratory performance. Further studies also with a larger sample are needed to confirm our findings.

## Conclusion

This study confirmed previous research present in the literature in which is reported that the quarantine period and the NPIs for COVID-19 caused a decrease in fitness levels in adolescents. However, this new school curriculum with a sport character established in Italy in high schools and called Sports High School (SHS) has shown not only to have positive effects in preserving the fitness levels of adolescents during the pandemic period, but also after 2-month of follow-up the students of SHS showed significant improvements in both lower and upper limb strength. This trend was not present in the THS group. School PE plays an important role in improving fitness levels in children and adolescents. However, our results suggest the need to increase hours per week of school PE as a strategy for improving fitness levels in adolescents.

## Data availability statement

The raw data supporting the conclusions of this article will be made available by the authors, without undue reservation.

## Ethics statement

The studies involving human participants were reviewed and approved by Independent Ethics Committee the University of Palermo (No. 77/2022 -Prot. 35307). Written informed consent to participate in this study was provided by the participants' legal guardian/next of kin.

## Author contributions

AB and APat: conceptualization. VG, FF, and MM: data collection. APat: data analysis. APat, VG, and MB: methodology. AB: supervision. PD and APal: visualization. APat: writing—original draft. VG and APal: writing—review and editing. All authors contributed to the article and approved the submitted version.

## Funding

The publication was made with the co-financing of the European Union - FESR or FSE, PON Research and Innovation 2014-2020 - DM 1062/2021.

## Conflict of interest

The authors declare that the research was conducted in the absence of any commercial or financial relationships that could be construed as a potential conflict of interest.

## Publisher's note

All claims expressed in this article are solely those of the authors and do not necessarily represent those of their affiliated organizations, or those of the publisher, the editors and the reviewers. Any product that may be evaluated in this article, or claim that may be made by its manufacturer, is not guaranteed or endorsed by the publisher.
